# Supplementary material for: Undersampled Diffusion-Weighted 129Xe MRI Morphometry of Airspace Enlargement: Feasibility in Chronic Obstructive Pulmonary Disease
Source: Diagnostics (Basel). 2023 Apr 19;13(8):1477. doi: 10.3390/diagnostics13081477 (PMC10137722; doi:10.3390/diagnostics13081477)
Supplement: Supplementary file 1 [file diagnostics-13-01477-s001.zip › Table S1.pdf]

Supporting Table S1. Participant listing of imaging measurements and estimates

	D′ cm²/s	Alpha	ADC cm²/s			*Lm*D μm			*L*m μm		
			FS	AF=2	AF=3	FS	AF= 2	AF=3	FS	AF=2	AF=3
**NS**	**.03 (.01)**	**.67 (.19)**	**.05 (.02)**	**.05 (.01)**	**.05 (.01)**	**140 (30)**	**130 (30)**	**140 (30)**	**280 (130)**	**300 (130)**	**300 (130)**
NS-1	.04 (.01)	.73 (.18)	.04 (.01)	.04 (.01)	.04 (.01)	150 (30)	150 (30)	150 (30)	310 (130)	340 (130)	340 (130)
NS-2	.03 (.02)	.59 (.21)	.05 (.02)	.05 (.02)	.05 (.02)	120 (40)	120 (40)	120 (40)	240 (140)	250 (150)	260 (150)
NS-3	.03 (.01)	.67 (.20)	.04 (.02)	.04 (.01)	.05 (.02)	140 (30)	140 (30)	140 (30)	260 (120)	290 (130)	290 (120)
NS-4	.04 (.01)	.69 (.19)	.05 (.02)	.05 (.01)	.05 (.01)	150 (30)	160 (30)	150 (30)	300 (130)	340 (130)	300 (110)
**COPD**	**.09 (.04)**	**.67 (.20)**	**.08 (.02)**	**.08 (.02)**	**.08 (.02)**	**190 (50)**	**190 (50)**	**190 (50)**	**560 (240)**	**570 (250)**	**580 (250)**
COPD-1	.12 (.05)	.51 (.24)	.10 (.02)	.10 (.02)	.10 (.02)	170 (60)	150 (60)	150 (60)	430 (270)	410 (270)	420 (270)
COPD-2	.08 (.03)	.76 (.18)	.08 (.02)	.08 (.02)	.08 (.02)	210 (50)	220 (50)	220 (50)	650 (240)	710 (250)	700 (230)
COPD-3	.08 (.03)	.69 (.21)	.07 (.02)	.07 (.02)	.07 (.02)	190 (50)	180 (50)	180 (50)	530 (240)	520 (250)	530 (250)
COPD-4	.09 (.05)	.63 (.22)	.08 (.03)	.08 (.03)	.08 (.03)	180 (50)	180 (50)	180 (50)	500 (250)	480 (270)	510 (270)
COPD-5	.09 (.03)	.77 (.14)	.08 (.02)	.08 (.02)	.08 (.02)	220 (40)	230 (30)	230 (30)	690 (210)	730 (180)	730 (180)

FS=Fully Sampled k-space; AF=Acceleration Factor; D′=apparent MRI diffusivity estimate; Alpha=MRI-derived heterogeneity index *Lm*D=MRI mean airway length scale estimate; *L*m=MRI mean linear intercept estimate. NS=Never-Smoker; COPD=ex-smoker with COPD; COPD-5=AATD patient
